# Microbiological Quality of Maize Silage in Relation to Agricultural Practices: A Four-Year Study

**DOI:** 10.3390/foods15091518

**Published:** 2026-04-27

**Authors:** Elżbieta Kukier, Łukasz Bocian, Monika Pytka

**Affiliations:** 1 School of Medical & Health Sciences, VIZJA University, 59 Okopowa Street, 01-043 Warsaw, Poland; 2 Department of Research Support, National Veterinary Research Institute, 57 Partyzantów Avenue, 24-100 Pulawy, Poland; lukasz.bocian@piwet.pulawy.pl; 3 Department of Biotechnology, Microbiology and Human Nutrition, University of Life Sciences, Akademicka 13, 20-950 Lublin, Poland; monika.pytka@up.lublin.pl

**Keywords:** maize silage, microbial safety, foodborne pathogens, *Clostridium*, *Listeria*, silage pH

## Abstract

Silage is a fundamental component of cattle feed, and its microbiological quality is critical for animal health and human safety. Improper ensiling conditions, such as oxygen exposure or inadequate acidification, can promote the growth of pathogens like *Listeria monocytogenes*, *Clostridium botulinum*, and *Bacillus cereus*. This study aimed to evaluate the microbial status of maize silages and identify pre-ensiling factors influencing its hygienic safety. Over a four-year period, 406 silage samples were collected from cattle farms across Poland. The research evaluated general hygiene indicators and screened for specific pathogens using standard culture methods, polymerase chain reaction toxotyping, and matrix-assisted laser desorption/ionization time-of-flight mass spectrometry. The impact of agricultural practices, including soil quality, organic fertilization, and microbial inoculation, was also analyzed. The analysis revealed that 32.1% of silages fell outside the reference pH range, indicating potential aerobic instability. While *Salmonella* and *Campylobacter* were not detected, *Clostridium* spp. were highly prevalent (81.0%), and *C. perfringens* was confirmed in 24.9% of samples. *Listeria* species occurred in 2.9% of silages, with *L. innocua* being the most frequent isolate. Statistical analysis showed that organic fertilization was significantly linked to specific *C. perfringens* toxotypes, though it did not increase the overall microbial burden. Conversely, microbial inoculation generally reduced the counts of several undesirable bacteria, although these differences were not statistically significant across all parameters. High pH values and significant contamination with *Clostridium*, *B. cereus*, and fungi remain critical challenges for silage safety. The results underscore the necessity for improved agricultural practices—specifically the minimization of soil and manure contamination during harvest—and the broader adoption of microbial inoculation to ensure the microbiological stability of fermented forage.

## 1. Introduction

Roughage forms the basis of cattle feed rations, accounting for up to 80% of total intake. Silage production in Poland has been steadily increasing since the second half of the 20th century, driven by the mechanization of forage harvesting [1]. This trend coincided with the growth of the cattle population—currently the third largest dairy herd in the European Union—further bolstered by the profitability of dairy production following the abolition of milk quotas [2,3]. High-quality silage is the most valuable conserved roughage for ruminants, characterized by low pH, high nutritional value, aerobic stability, palatability, and microbiological safety [4,5]. Maize (*Zea mays* L.) silage (MS) is a moist fodder produced from chopped whole maize plants preserved through ensiling. The microbial composition of ready-to-feed MS is influenced by the type and quantity of epiphytic microflora present on the plants prior to ensiling, the use of inoculants, and secondary contaminants introduced during the process, as well as storage, preparation, and feeding conditions [6,7]. Microbial proliferation is further promoted by the high water activity of the forage, its buffering capacity, and the availability of water-soluble carbohydrates [8]. The microbiological safety of MS is maintained by an anaerobic environment and the fermentation products of lactic acid bacteria (LAB), which lower the pH to suppress the growth of undesirable microorganisms [9]. However, if fermentation is compromised by insufficient acidification or oxygen exposure in the silo, MS becomes highly susceptible to aerobic deterioration and microbiological hazards [10]. Notable examples include *Escherichia* (*E.*) *coli*, *Listeria* (*L.*) *monocytogenes*, *Bacillus* (*B.*) *cereus*, *B. licheniformis*, or *Clostridium* (*C.*) *botulinum*, with minimum growth pH limits of 3.4, 4.2, 4.3, 4.5, and 4.6, respectively [11,12,13,14].

Furthermore, diet has a profound impact on the gut microbiota and directly affects host health in terms of metabolism and immunity [15,16]. Cattle fed poor-quality forage become immunocompromised, which increases the prevalence of mastitis, diarrhea, hoof diseases, metabolic disorders, and reproductive failure. This also directly affects the immunity of newborn calves, making them more susceptible to infections [17,18,19,20,21,22,23,24].

In addition, poor-quality silage serves as a source of microbiological hazards in food of animal origin, thereby posing a risk to human health [25,26]. Several microbial hazards may be present in poor-quality silage, including pathogenic species of the *Listeria*, *Clostridium*, and *Bacillus* genera, members of the *Enterobacteriaceae* family, and toxigenic fungal species [27,28,29,30]. Since numerous investigations have identified ruminants as reservoirs of *Campylobacter* spp. and food products of bovine origin as potential sources of human foodborne campylobacteriosis, the role of forage in the transmission of this pathogen within the dairy farm environment remains unclear and requires further investigation [31,32].

The microbial status of silage is assessed using general hygiene indicators. The enzymatic activity of microorganisms reduces the nutritional value of feed and causes adverse organoleptic changes. Counts of the family *Enterobacteriaceae* reveal the fecal contamination of forage, as well as environmental contaminants, as some species in this family are known plant pathogens or inhabitants of soil and water [33,34]. *Clostridium* and *Bacillus* genera, which comprise spore-forming bacteria, are commonly found in soil, where they are involved in organic matter decay; they are also native inhabitants of the gastrointestinal tracts of insects and warm-blooded animals [35]. Due to their toxigenic potential and the resistance of their spores to harsh environmental conditions, they pose a serious quality and safety concern for producers of moist forage and food of animal origin. Furthermore, fungal contamination of forage poses a risk of animal and human mycotoxicosis, fungal infections, and allergies.

Furthermore, Directive 2003/99/EC on the monitoring of zoonoses and zoonotic agents requires Member States to collect, evaluate, and report data on zoonotic agents, and to monitor the stages of the food chain most appropriate to the agents concerned, including primary production. Taking these factors into account, the present study was designed to assess the microbial status of MS collected from cattle farms, including the prevalence of zoonotic agents, as well as pre-harvest and ensiling factors such as farm characteristics, maize cultivation practices, and the ensiling process. It was hypothesized that certain agricultural practices significantly affect the hygienic quality of maize silage.

## 2. Materials and Methods

### 2.1. Experimental Design and Sampling

**Figure 1:**
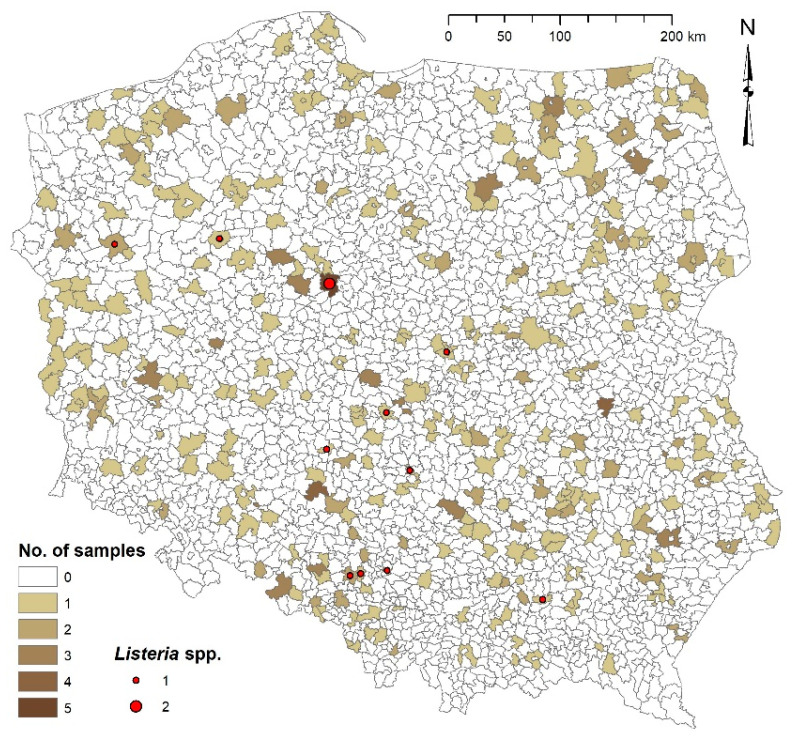
Geographical distribution of MS samples across Polish municipalities. The intensity of the background color indicates the number of samples collected in each municipality. Red dots indicate locations where *Listeria* spp. were isolated, with dot size corresponding to the number of isolates.

A total of 406 MS samples were collected from Polish cattle farms, including 107 samples in 2014, 92 in 2015, 107 in 2016, and 100 in 2017. Random sampling was conducted across the country by Veterinary Officers from January to November, with six samples collected per year in each of the 16 voivodeships. Figure 1 shows the number of samples collected in the individual municipalities of the voivodeships. The MS collection procedure involved sampling into sterile plastic bags from bunker or trench silos after removing the surface layer and any areas with visible mold, while ensuring that oxygen exposure was kept to a minimum. In accordance with good microbiological practice, aseptic techniques were applied to prevent secondary contamination. Samples were frozen immediately to inhibit microbial activity and delivered within 24 h in insulated containers with ice to the laboratory of the Department of Hygiene of Animal Feedingstuffs at the National Veterinary Research Institute (NVRI) in Puławy, Poland. There, they were stored at −20 °C until analysis [36]. At least 24 h prior to testing, the samples were thawed at 2–8 °C. Each sample was accompanied by a questionnaire designed to gather insights into farm characteristics, maize cultivation practices, and the silage-making process. Data collected included: cattle herd size, the farmer’s experience in forage ensiling (in years), soil quality at the harvest site, application of organic fertilizers and herbicides, use of silage inoculants, silage maturity (the period between ensiling and sampling), and herd health history, specifically the past incidence of zoonotic diseases such as listeriosis and botulism.

### 2.2. Measurement of pH Value

To measure the pH, 100 g of MS was weighed into a sterile filter stomacher bag and mixed with 100 mL of double-distilled water. The sample was then homogenized for 2 min using a stomacher (MIX2, AES Laboratoire; Combourg, France) and stored at 2–8 °C overnight. Approximately one hour before measurement, the sample was equilibrated to room temperature. Prior to the pH reading, the electrode of the CG 843P Laboratory pH meter (SCHOTT; Mainz, Germany) was rinsed with distilled water and calibrated. The electrode was then immersed in the sample filtrate, and the pH value was recorded. The reference pH range for maize silage was defined as 3.70 to 4.29 [37].

### 2.3. Microbiological Analyses

Microbiological analyses were performed according to the standard culture methods detailed in Table 1 to detect the presence of *Salmonella* spp., *Clostridium* spp., *L. monocytogenes*, and *Campylobacter* spp. Additionally, counts were determined for total plate count (TPC), aerobic mesophilic bacteria (AMB), fungi, anaerobic spore-forming bacteria (ASFB), the *Enterobacteriaceae* family, *E. coli*, presumptive *B. cereus*, *C. perfringens*, lactic acid bacteria (LAB), and coagulase-positive staphylococci (CoPS) [38,39,40,41,42,43,44,45,46,47,48,49]. Forage samples, initial suspensions, and decimal dilutions were prepared in accordance with ISO 6887-4:2017 [50]. Quantitative tests were conducted in duplicate. To validate experimental conditions, negative and positive controls were established simultaneously using reference strains.

### 2.4. Typing of Clostridium spp. Isolates by PCR Methods

*C. perfringens* isolates obtained via the standard culture method were toxotyped using a multiplex polymerase chain reaction (mPCR) to detect the presence of toxin genes: *cpa* (α toxin), *cpb* (β), *cpb2* (β2), *etx* (ε), *iap* (ι), and *cpe* (enterotoxin) [51]. This method was slightly modified; template DNA was extracted from thermolysed overnight cultures of *C. perfringens* grown on Willis-Hobbs agar and incubated at 37 °C under anaerobic conditions [52]. The mPCR was performed using a T-1 Thermoblock thermocycler (Biometra, Göttingen, Germany). Bacterial isolates suspected of being botulinum neurotoxin (BoNT)-producing *Clostridia* were identified by real-time PCR targeting the nontoxic nonhemagglutinin encoding gene (*ntnh*), which is characteristic of all BoNT-producing *Clostridia* [53]. DNA was extracted using the Genomic MINI AX Bacteria kit (A&A Biotechnology, Gdańsk, Poland) following the manufacturer’s protocol. The real-time PCR assays were performed on a LightCycler 2.0 System (Roche, Rotkreuz, Switzerland).

### 2.5. Identification of Bacteria by MALDI-TOF MS

Unknown microorganisms were identified using matrix-assisted laser desorption/ionization time-of-flight mass spectrometry (MALDI-TOF MS) with a Bruker MALDI Biotyper (Bruker, Bremen, Germany). Following the manufacturer’s ethanol-formic acid extraction protocol, a loopful of overnight culture grown on horse blood agar was transferred into a microcentrifuge tube containing 300 µL of ultrapure water and mixed thoroughly; then, 900 µL of 100% ethanol (Sigma-Aldrich, Schnelldorf, Germany) was added. The cell suspension was centrifuged at 13,000× *g* for 2 min, and the supernatant was decanted. After a second centrifugation, residual ethanol was removed by pipetting. To the air-dried pellet, 10–40 µL of 70% formic acid (Sigma-Aldrich) was added—depending on the inoculum size—followed by thorough pipetting and the addition of an equal volume of acetonitrile (Sigma-Aldrich). The sample was centrifuged at 13,000× *g* for 2 min, and 1 μL of the supernatant was deposited onto a spot on the MALDI target plate (Bruker Daltonics GmbH, Bremen, Germany). Once dried, the sample was overlaid with 1 μL of HCCA matrix solution (10 mg/mL α-cyano-4-hydroxycinnamic acid; Bruker Daltonics, Bremen, Germany) dissolved in a standard solvent (50% acetonitrile, 47.5% water, and 2.5% trifluoroacetic acid; Sigma-Aldrich, Schnelldorf, Germany). Finally, the air-dried sample spots were analyzed using an Autoflex Speed system (Bruker, Bremen, Germany) operated by flexControl 3.3 software. Identification was performed using MBT Compass 4.1.70 software with the MBT Library BDAL-6903 reference database [54]. Each isolate was analyzed in duplicate. Score values of 2.0–3.0 indicated reliable identification at the species level; scores of 1.7–1.99 were considered reliable at the genus level, while scores <1.70 were interpreted as unreliable.

### 2.6. Calculations and Statistics

Results of microbial qualitative analyses (presence of microorganisms) were expressed as prevalence in a percentage. The quantitative analyses (counts) were calculated in colony forming units per gram (cfu/g) and subsequently transformed using the formula *y* = log_10_(*x*), where *x* denotes the observed microbial count expressed in cfu/g, prior to statistical analysis. Since all quantitative microbiological results were greater than zero, no constant was added before transformation. No observations were excluded as outliers, and all samples meeting the predefined study inclusion criteria were retained for statistical analyses. The results of *Clostridium* spp. presence were calculated in titer, known as the minimum weight of a sample (in grams) where microorganisms were detected. For graphical representation, the results were transformed into a range of microorganisms where a titer less than 0.1 means below 10 cfu/g (below 1 log_10_), a 0.1 titer ranges from 10 to 99 cfu/g (1 log_10_), and a 0.01 titer ranges from 100 to 999 cfu/g (2 log_10_), and so on. The microbial load was analyzed both in silages with correct pH as well as in samples with incorrect pH values. The influence of atmospheric conditions on the microbial status of silages was recognized, taking into account average annual rainfall (2014—644.3 mm, 2015—501.2, 2016—701.2, 2017—794.16) and mean annual temperature (2014—8.4 °C, 2015—8.4, 2016—8.1, 2017—7.75) in Poland each year.

The normality of data distribution was formally assessed using the Shapiro–Wilk test. The qualitative parameters of microbial samples (MSs) were compared using χ^2^ tests, and quantitative variables were analyzed using the non-parametric Mann–Whitney U test due to the lack of normal distribution of results. Dichotomous variables were also analysed using logistic regression models, and the results are reported as odds ratios (OR) with corresponding 95% confidence intervals (CI) and *p*-values. The significance of the independent variables was assessed using the Wald test. Regression coefficients were estimated using the maximum likelihood method. The fit of the model to the data was assessed using the likelihood ratio test (LR statistic). The Kruskal–Wallis test and the post hoc multiple comparison test were used to compare values across individual years of the survey. The strength and direction of associations between farm characteristics and silage microbial parameters were measured using Spearman’s rank correlation coefficient. For the purposes of this paper, absolute values of R from 0.1 to 0.3 were regarded as weak, 0.3–0.5 as moderate, and 0.5–1.0 as strong correlations. For statistical data analysis, TIBCO Software Inc. (Palo Alto, CA, USA) Statistica (data analysis software system), version 13 (2017), was used. A *p*-value ≤ 0.05 was considered statistically significant in all analyses. The geographical distribution of samples and spatial visualization of microbiological findings shown in Figure 1 were prepared using ArcGIS Desktop 10.4.1 software (Esri Inc., Redlands, CA, USA).

## 3. Results

### 3.1. Farm Characteristics

The MS samples were collected from farms with livestock numbers ranging from 6 to 2500. Farmers’ experience in maize ensiling ranged from 1 to 70 years. The green fodder was sourced from fields spanning agricultural soil classes I through VI, categorized as follows: class I (excellent), class II (very good), class III (good), class IV (medium), class V (poor), and class VI (very poor). The use of organic fertilizers was declared by 74.9% (304/406) of farmers, including manure or slurry used by 74.4% (302/406) and poultry litter by 1% (4/406) of farms. None of the farmers fertilized their maize crops with biogas digestate. The maize crops were treated with herbicides to eliminate unwanted plants in 87.7% (356/406) of cases. Microbial silage inoculant was added during the ensiling process in 39.6% (161/406) of cases, including 43% in 2014, 34.8% in 2015, 37.4% in 2016, and 43% in 2017. The maturity of the silage at the time of sampling varied widely, ranging from 1 to 24 months. Each batch of tested silage was declared safe by farmers, and no animal diseases were diagnosed due to unhygienic silage before sampling. However, the survey data showed the incidence of clinical listeriosis at two farms (0.5%) and botulism at one farm (0.2%) in the past. The surveyed silages were not visibly mold-infested upon macroscopic evaluation by laboratory staff.

### 3.2. PH Value of Maize Silages

The pH of the MS samples ranged from 2.1 to 7.05. Overall, 67.9% of the silages (276/406) fell within the reference pH range, while the remainder were outside this range. Specifically, 35.9% of the MS samples had a pH below 4.0, 59.6% had a pH between 4.0 and 5.0, and 4.4% had a pH exceeding 5.0.

### 3.3. Prevalence of Bacteria

*Listeria* species were detected in 2.9% (12/406) of the total MS samples, including 3.3% (9/276) of silages with correct pH and 2.3% (3/130) with incorrect pH (Figure 1). Members of the *Listeria* genus were found in MS samples with a pH range of 3.7 to 6.8. The prevalence of individual *Listeria* species across all samples was: 1.5% *L. innocua*, 1% *L. seeligeri*, and 0.25% each for *L. monocytogenes* and *L. ivanovii*. Of the 12 *Listeria* isolates, six were *L. innocua* (50%), four were *L. seeligeri* (33.3%), and one each was *L. monocytogenes* (8.3%) and *L. ivanovii* (8.3%). While all four *Listeria* species were found in silages with correct pH, samples with incorrect pH contained only *L. innocua* and *L. seeligeri*.

*Clostridium* spp. were detected in 81% (329/406) of the total MS samples, comprising 78.3% of those with correct pH and 86.1% with incorrect pH. *C. perfringens* was confirmed in 24.9% (101/406) of total samples, 21.4% with correct pH, and 30.8% with incorrect pH. No *C. botulinum* strains were identified, as the *ntnh* gene of BoNT-producing *Clostridia* remained undetectable in all isolates. *Salmonella* spp. and *Campylobacter* spp. were not detected in any of the tested samples. The prevalence of *Enterobacteriaceae*, *E. coli*, and *B. cereus* was 2.9%, 2%, and 23.1%, respectively, with lower occurrences in MS with correct pH (2.2%, 1.8%, 20.6%) compared to incorrect pH forage (4.6%, 2.3%, 27.7%). Identification of unknown *Bacillus* isolates revealed the presence of *B. weihenstephanensis*, *B. mycoides*, and *B. thuringiensis*. No CoPS were found in the 406 silages analyzed; only one sample (0.2%) with a pH of 6.6 tested positive for coagulase-negative *Staphylococcus lentus*.

### 3.4. Toxotype of C. Perfringens Isolates

Among the 101 *C. perfringens* isolates analyzed, the following toxotypes were identified: A (98.1%), D (0.9%), E (0.9%), and F (0.9%). Isolates from silage with correct pH were classified as types A (96.6%), D (1.7%), and F (1.7%), while those from silage with incorrect pH were classified as types A (97.5%) and E (2.5%). The gene encoding the beta2-toxin was detected in 39.6% of all *C. perfringens* isolates, including 32.2% from silages with correct pH and 52.5% from those with incorrect pH. The presence of the *cpb2* gene was confirmed in isolates of type A (39.4%) and type D (100%).

### 3.5. Count of Hygiene Indicators

The mean values for AMB and TPC in MS samples with correct pH were approximately 5.0 log_10_ cfu/g, with ranges varying from 1 to 9 log_10_ cfu/g. While the ranges for LAB and fungi counts were identical, the mean value for fungi was nearly half that of LAB. The average contamination levels for the *Enterobacteriaceae* family, *E. coli*, *C. perfringens*, and presumptive *B. cereus* were around 1 log_10_ cfu/g. However, maximum values reached up to 2.6 log_10_ cfu/g for *C. perfringens*, 3.4 log_10_ spores/g for presumptive *B. cereus*, 4.7 log_10_ cfu/g for *E. coli*, and 6.2 log_10_ cfu/g for *Enterobacteriaceae*. The average level of ASFB contamination was estimated at 2.5 log_10_ cfu/g, with these bacilli ranging from 1 to 5 log_10_ cfu/g. Median values were generally lower than the means, except for *E. coli* and *C. perfringens* counts. Table 2 presents the microbial counts in MS samples with correct pH.

The MS samples with incorrect pH exhibited a greater microbial load than those with correct pH, which was reflected in higher mean values (up to 0.7 log_10_ cfu/g) and elevated upper limits for microorganism ranges (up to 1.7 log_10_ cfu/g). The *Enterobacteriaceae* family and *E. coli* were exceptions to this trend. Differences were also noted in the distribution of samples across different levels of microbial load. Higher contamination levels were confirmed for fungi, *Clostridium* spp., *C. perfringens*, and *B. cereus*. Median values were correspondingly lower than the means, except for LAB and *Clostridium* counts, which were estimated to be higher than the averages. Table 3 details the microbial counts in MS samples with incorrect pH.

The count of microorganisms in total silages was 1.6–9.7 log_10_ cfu/g of TPC, 1.8–9.7 log_10_ cfu/g of AMB, 1–8.9 log_10_ cfu/g of fungi, 1–9.3 log_10_ cfu/g of LAB, 1–6.2 log_10_ cfu/g of *Enterobacteriaceae* family, 1–4.7 log_10_ cfu/g of *E. coli*, 1–5 log_10_ cfu/g of *Clostridium*, 1–4.2 log_10_ cfu/g of *C. perfringens*, and 1–3.4 log_10_ cfu/g of *B. cereus*. The percentage of silages with different levels of microorganisms in total MSs is demonstrated in Table 4.

The χ^2^ test demonstrated a statistically significant association between the pH category of MS and the presence of *C. perfringens* (*p* = 0.0175). Logistic regression analysis showed that *C. perfringens* presence in MSs reduced the odds of a correct pH range by almost half (OR = 0.57; 95% CI: 0.36–0.91; *p* = 0.0182). In turn, the analysis of quantitative variables showed statistically significant differences between silages with correct and incorrect pH in terms of microbial load, including TPC (*p* = 0.0001), the count of AMB (*p* = 0.0312), fungi (*p* = 0.0050), LAB (*p* = 0.0036), and *C. perfringens* (*p* = 0.0047), as well as silage maturity (*p* = 0.0318). Statistically significant differences were shown in TPC between the years 2014 and 2016 (*p* = 0.0013) and 2014 and 2017 (*p* = 0.0045); in the count of fungi between the years 2014 and 2015 (*p* = 0.0213), 2014 and 2016 (*p* = 0.0265), and 2014 and 2017 (*p* = 0.0119); in the count of LAB between the years 2014 and 2015 (*p* < 0.0001), 2014 and 2016 (*p* < 0.0001), and 2014 and 2017 (*p* < 0.0001); in the count of *Clostridium* between the years 2014 and 2016 (*p* = 0.0002) and 2014 and 2017 (*p* < 0.0001); and in the count of *B. cereus* between the years 2014 and 2015 (*p* = 0.0045) and 2015 and 2017 (*p* = 0.0020). Furthermore, the study demonstrated significant differences in silage pH between the years 2014 and 2017 (*p* < 0.0001), 2015 and 2017 (*p* < 0.0001), and 2016 and 2017 (*p* < 0.0001). Significant differences were also observed in average annual rainfall between all surveyed years (all *p* < 0.0001) and in mean annual temperature for all year-to-year comparisons except 2014 versus 2015 (all *p* < 0.0001). Other indicators were not statistically significant.

### 3.6. Effect of Organic Fertilization

Silages prepared from organically fertilized maize crops showed a pH range of 2.10 to 7.05, with a mean and median of 4.09. In contrast, for non-fertilized forages, the pH ranged from 3.10 to 6.87, with a mean of 4.19 and a median of 4.07. The prevalence of microorganisms in organically fertilized MS samples was 2.6% for *Listeria* spp., 81.6% for *Clostridium* spp., 26% for *C. perfringens*, 2.6% for the *Enterobacteriaceae* family, 1.6% for *E. coli*, and 24.3% for *B. cereus*. In non-fertilized MS, these values were 3.9%, 79.4%, 21.6%, 3.9%, 2.9%, and 19.6%, respectively. Additionally, one case of botulism (0.3%) and two cases of listeriosis (0.6%) were diagnosed on farms using organic fertilization, whereas these diseases were not recorded on farms that did not use organic fertilizers. The mean contamination levels for AMB, *Clostridium*, and *B. cereus*, the median values for TPC, AMB, and *Clostridium*, and the upper limits for fungi, *Enterobacteriaceae*, *E. coli*, and *B. cereus* were slightly higher in silages from organically fertilized crops. However, organic fertilization did not result in statistically significant differences in the microbial load of the MS samples tested. A statistically significant link was only observed between organic fertilization and toxotypes of *C. perfringens* (*p* < 0.0001).

### 3.7. Effect of Silage Inoculation

Microbiologically inoculated MSs showed a pH range of 2.10 to 6.87, with a mean of 4.10 and a median of 4.06. In contrast, MSs without inoculants had values of 2.25 to 7.05, with a mean of 4.12 and a median of 4.10. The prevalence of bacterial indicators in microbiologically inoculated MSs was 1.9% for *Listeria* spp., 79.5% for *Clostridium* spp., 22.4% for *C. perfringens*, 2.5% for the *Enterobacteriaceae* family, and 1.2% for *E. coli*, compared to non-inoculated silages, where the above parameters were 3.7%, 82%, 26.5%, 3.3%, and 2.4%, respectively. The prevalence of *B. cereus* showed an opposite trend, increasing to 25.5% after inoculation compared to 21.6% in non-inoculated MSs. Inoculation also reduced the counts of TPC, AMB, fungi, LAB, *Enterobacteriaceae*, *E. coli*, *Clostridium*, and *C. perfringens*, but the *B. cereus* count was higher than in non-inoculated feeds. The above divergences in microbial load of MSs were not statistically significant. Significant associations were confirmed only for experience in ensiling (*p* = 0.0315), herd size (*p* < 0.0001), silage maturity (*p* = 0.0057), and herbicide application, with the latter being approximately twice as likely on farms using microbial inoculation (OR = 2.03; 95% CI: 1.04–3.94; *p* = 0.0350). Table 5 shows the differences in microbial load of inoculated and non-inoculated MSs and organically fertilized and non-fertilized MSs.

### 3.8. Correlation of Parameters

**Figure 2:**
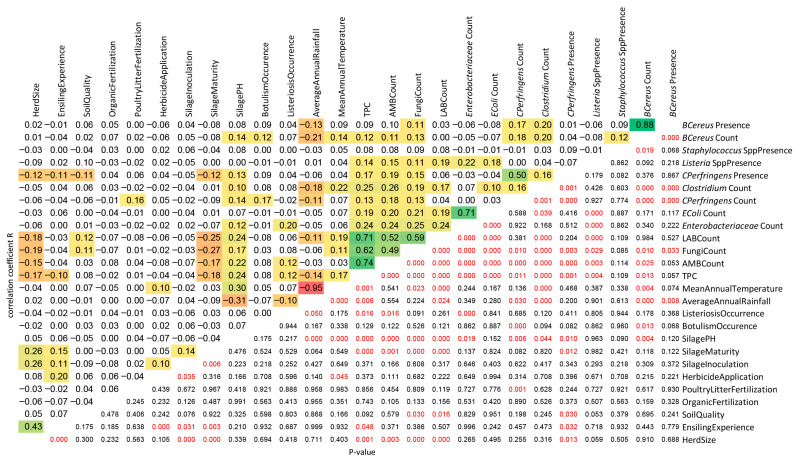
Spearman’s correlation matrix showing relationships among all analyzed farm, environmental, and microbiological variables. Correlation coefficients are presented in the upper left corner, while the statistical significance of the correlations is displayed in the lower right corner. *p*-values marked in red indicate statistically significant correlations, and statistically significant correlation coefficients are highlighted using a red–yellow–green color scale ranging from −1 (negative correlation) to +1 (positive correlation).

Inoculants were more readily used by farmers of larger herds experienced in ensiling, and silages with lower counts of TPC, AMB, fungi, LAB, and *C. perfringens* were observed on farms with larger herds (Figure 2). Silage inoculation was positively correlated with herbicide application and mean annual temperature. Farmers’ ensiling experience was positively correlated with herbicide application and negatively correlated with TPC and *C. perfringens* prevalence. The worst soil class for maize cultivation was correlated with a higher count of fungi and LAB in silage. In contrast, better soil quality favored the presence of *C. perfringens*. Poultry litter fertilization was positively correlated with *C. perfringens* contamination of forage. Older silages had lower loads of TPC, AMB, fungi, *C. perfringens*, and LAB. Silage pH was positively correlated with TPC, AMB, fungi, LAB, *Enterobacteriaceae*, *C. perfringens*, *Clostridium*, *B. cereus*, and mean annual temperature. A moderate negative correlation was identified between silage pH and average annual rainfall. Bovine botulism occurred more often when MS was more heavily burdened by *C. perfringens* and *B. cereus*. In contrast, a higher count of *Enterobacteriaceae*, TPC, or AMB in silage increased the occurrence of listeriosis. Rainfall was negatively correlated with silage contamination by *B. cereus*, *Clostridium*, *C. perfringens*, LAB, TPC, and temperature. A weak positive correlation was found between temperature and the load of silage by *B. cereus*, *Clostridium*, LAB, fungi, and TPC. The count of general hygiene indicators such as TPC, AMB, and fungi was positively correlated with silage contamination by the *Enterobacteriaceae* family, including *E. coli*, the *Clostridium* genus, including *C. perfringens*, *Listeria* species, and *B. cereus*. A weak positive correlation was observed between the count of LAB and silage burden by *Enterobacteriaceae*, *Clostridium*, and *Listeria* species. A positive dependence was found between the count of the *Enterobacteriaceae* family and the incidence of *Listeria*, the occurrence of listeriosis, and the pH of silage. Similarly, the count of *E. coli* was positively linked to the prevalence of *Listeria* species and the count of general hygiene indicators. The contamination of MS by anaerobic spore-formers *Clostridia*, including *C. perfringens*, was correlated with contamination by *B. cereus*. The incidence of *Listeria* was favored by a higher count of *Enterobacteriaceae* and general hygiene indicators. A higher load of MS by *B. cereus* was positively associated with a higher count of *Clostridia*, TPC, AMB, fungi, as well as higher silage pH and mean annual temperature.

## 4. Discussion

The impact of feed on animal health, the safety of food of animal origin, and human health is indisputable. Consequently, understanding the microbial status of silage and the pre-harvest and ensiling factors associated with this forage is essential for identifying critical control points and implementing preventive measures to minimize hazards during primary production and subsequent stages of the food chain. To date, research on the microbial status of MS has primarily focused on the effects of silage additives or investigations into listeriosis and botulism outbreaks where silage was the putative source of the pathogen [55,56,57,58,59,60,61,62,63]. There are relatively few data available on the prevalence of microbial hazards in MS not directly associated with feed-borne disease [64]. The present research was based on random samples collected nationwide over four consecutive years to ensure that the results were as reliable and representative as possible.

The primary concern revealed in our study relates to suboptimal pH values in every third MS sample, which compromises the safety of the forage. An excessively high pH indicates poor fermentation, often resulting from low moisture content due to delayed harvesting or drought. Conversely, an excessively low pH leads to overly vigorous fermentation and a loss of nutrients, typically caused by early harvesting [37]. Consequently, poorly fermented silage is aerobically unstable due to insufficient acid production, offering weak protection against the uncontrolled proliferation of undesirable microorganisms. It has been reported that more than 35% of silages from cattle farms experiencing listeriosis outbreaks had an insufficiently low pH [65]. According to the pH-based quality classification—categorized as very good (pH 3.2–4.2), good (4.2–4.5), moderate (4.5–4.8), and poor (>4.8)—the tested MS samples were classified as 67.2% very good, 21.2% good, 3.7% moderate, and 5.4% poor quality [66]. Although samples were taken from the silo core, the pH range recorded in our research was slightly broader than the 3.2 to 6.6 range reported by other authors [67,68,69,70,71,72,73,74]. For comparison, poor fermentation quality affected 0% to 5% of maize silages in a descriptive evaluation conducted across various German regions, including Lower Saxony, Schleswig-Holstein, Brandenburg, Mecklenburg-West Pomerania, Saxony-Anhalt, Thuringia, and Bavaria [69].

The analyzed MS samples served as a reservoir for well-known pathogens, such as *L. monocytogenes* and *L. ivanovii*, as well as less virulent species, including *L. innocua* and *L. seeligeri*, which are sporadically reported as causative agents of human infection [75,76]. The recorded prevalence of *Listeria* spp. was similar to that reported in Latvia (3.1%), but lower than in Turkey (6.6%), the U.S. (10.8%), Finland (22.7%), Spain (33.7%), and France (62%) [70,77,78,79,80,81]. Similarly, the prevalence of *L. monocytogenes* (0.25%) was notably lower than the 3%, 4.7%, 6%, 10%, 16%, 17%, 28.6%, or 39% reported in other studies [70,77,78,79,81,82,83,84,85]. The low prevalence of *Listeria* spp. in the MS samples may stem from the low environmental occurrence of these bacteria in soil, EU regulations limiting manure application in maize cultivation and climatic conditions in Poland [86]. A significantly higher prevalence of *L. monocytogenes*, ranging from 25% to 46%, has been observed on farms where cattle suffered from listeriosis [65]. In the present study, the prevalence of *L. innocua* in Polish MSs was lower than the 6%, 19.3%, and 30% reported in Irish, Spanish, and U.S. studies, respectively [81,83,85]. The recorded prevalence of *L. seeligeri* was comparable to Spanish observations, where it amounted to 1.2% [81]. Interestingly, our study identified *L. ivanovii*, albeit rarely, in contrast to its absence in other reports [81,85]. We did not detect other *Listeria* species, such as *L. welshimeri* or *L. grayi*, which were noted in a Spanish survey [81]. The predominant species within the *Listeria* genus was *L. innocua*, consistent with Spanish, Irish, and U.S. studies, where it accounted for 57.1%, 66.6%, and 71.4% of isolates, respectively [70,81,85]. *L. seeligeri* was the second most common species identified in our survey.

Despite the dominance of less virulent species, the presence of any *Listeria* indicates the potential for highly pathogenic species to grow within the silage environment, as well as the risk of aerobic spoilage in *Listeria*-positive forage. In our study, *Listeria*-positive MS samples had a pH range of 3.7 to 6.8, which differs slightly from other surveys reporting ranges of 3.8–5.2 or 4.47–6.97 [81,85]. A second concern relates to the environmental tolerance of most *Listeria* isolates (75%) obtained from MS with a correct pH, and the lower pH limit (3.7) at which the bacteria survived—this is below the thresholds previously reported. This observation confirms the ability of *Listeria* species to mount an acid tolerance response (ATR), which enhances survival at low pH after exposure to mildly acidic conditions. Consequently, the prevalence of *Listeria* spp. did not correlate with silage pH in our study, whereas other surveys have consistently shown a positive correlation between these factors [80,81,87].

Although the silage environment is generally not conducive to the growth of *Clostridia*, their prevalence was common and higher in silages with incorrect pH. Half of the MS samples exhibited contamination levels above 2 log_10_ cfu/g, which is considered a cause for concern, while 21% of samples exceeded 3 log_10_ cfu/g, a dangerously high level. Other surveys report *Clostridia* counts ranging from below 3 log in the central part of a silage bunker to above 5 log spores/g in peripheral areas, with specific reported values of 1.01–2.68 log_10_ cfu/g, 2.8–3.12 log_10_ cfu/g, 2.34 ± 0.87 log_10_ MPN/g, 10^2^–10^7^ cfu/g, and 1.7–1.84 log_10_ MPN/g [7,26,72,88,89,90]. Czech researchers suggest that the maximum count of *Clostridia* spores in silage should not exceed 5 × 10^3^ cfu/g [91,92]. Based on this guideline, 79% of the tested MS samples maintained an acceptable level of ASFB. *Clostridia* are typically intolerant of pH values below 4.5, and a positive correlation between silage pH and *Clostridia* or *C. perfringens* counts was observed in our study (Figure 1) [93]. Notably, the presence of *C. perfringens* in MS had a significant negative impact on pH stability, with the strength of this effect being approximately twice that of other factors. The addition of LAB inoculants significantly reduced *Clostridia* counts, confirming a negative correlation between these parameters [7]. Furthermore, an increase in average annual rainfall in Poland noticeably reduced most microbial indicators, likely because rain washes soil and manure particles from the plants. Moreover, we demonstrated a distinct positive correlation between the counts of *Clostridia* species and *B. cereus*, which could serve as a predictive tool for MS contamination when only one indicator is monitored.

The species associated with silage is *C. perfringens*, present in every fourth tested MS, and its higher prevalence in silages with incorrect pH was shown. A similar level of *C. perfringens* contamination was noted in our study of silages with correct pH and in another Polish study where the ranges were 1–2.6 log_10_ cfu/g and 0.5–2.3 log_10_ cfu/g, respectively [74]. According to the literature, recommended *C. perfringens* levels in MS are: optimum < 50 spores/g, upper limit 20,000 spores/g, mean 500–700 spores/g, and median 50–100 spores/g [94]. With respect to these values, 95.3% of surveyed MSs demonstrated an optimum *C. perfringens* count, no MS exceeded the upper limit, and the mean and median values were 61 and 10 cfu/g, respectively. The predominant type was *C. perfringens* type A, the primary culprit in hemorrhagic bowel syndrome and acute enterotoxemia, mainly affecting dairy cattle [95]. Sporadically isolated were *C. perfringens* type D and E, responsible for enterotoxemia in domestic ruminants. Additionally, analyzed MSs were identified as a potential source of *C. perfringens* type F, formerly referred to as enterotoxigenic *C. perfringens*, the cause of food poisoning and antibiotic-associated diarrhea in humans and enteritis in animals [96,97,98]. Almost 40% of investigated *C. perfringens* isolates were *cpb2* gene-positive, while German scientists reported this rate at 23.1% of feed isolates [99]. Interestingly, our survey demonstrated a significantly higher prevalence of β2-toxigenic *C. perfringens* isolates in MS of incorrect pH versus isolates from correct pH forages. To the authors’ knowledge, this is the first report on the toxotyping of *C. perfringens* isolates derived from MSs of both correct and incorrect pH.

An extremely toxic anaerobe, *C. botulinum*, does not ferment carbohydrates and normally does not grow in well-fermented silage. However, outbreaks of bovine botulism due to BoNT-producing *Clostridia* in silage, often with a high mortality rate, are frequently reported [21,100,101]. In most outbreaks, the occurrence of BoNT-producing *Clostridia* and BoNTs was associated with the presence of bird carcasses from poultry litter or small animals accidentally killed during harvesting [102]. Although one farm had a history of cattle botulism, our study found no correlation between the outbreak and the fertilization of maize crops with poultry litter, as the implicated farm used only manure as fertilizer.

The ability of *Campylobacter* to colonize, its oxidative stress defenses, and its ability to survive in a wide variety of animal species and habitats make the microorganism extremely difficult to control [103]. Dairy products are considered the second major potential vehicle for transmitting *Campylobacter* spp. to humans, and these seemingly fastidious microaerophilic bacteria are present in cattle feces and manure, the dairy farm environment, soil, water, and edible parts of crops [104,105,106,107]. Notwithstanding the foregoing, our study did not detect the presence of *Campylobacter* spp. in MSs and did not reveal MS to be the source or vehicle of this pathogen along the production chain of dairy products. Our observation is consistent with the results of other researchers who did not detect *Campylobacter* species in MSs either [83].

Concern regarding *Enterobacteriaceae* contamination stems primarily from the potential presence of pathogens affecting both livestock and humans, such as *E. coli* or species within the *Salmonella*, *Shigella*, *Yersinia*, *Klebsiella*, *Cronobacter*, *Enterobacter*, and *Proteus* genera [108,109]. As facultative anaerobes, *Enterobacteriaceae* are the most significant competitors for nutrients against lactic acid bacteria (LAB). Their presence in silage often originates from slurry-fertilized forage crops or environmental contamination, and elevated levels typically indicate poor fermentation quality [110,111]. This family is generally sensitive to a pH below 4.5. Consequently, a high count (approximately 10^5^ cfu/g) in MS is typically observed only during the initial days of ensiling, thanks to the rapid acidification of this readily fermentable crop [112,113]. This trend was further evidenced by the negative correlation between MS maturity and the *Enterobacteriaceae* count in our analyses. However, survival and renewed growth of *Enterobacteriaceae* can occur if oxygen infiltrates the silage, leading to counts exceeding 10^8^ cfu/g as the pH increases during aerobic deterioration [11]. Against this background, the prevalence and contamination levels of *Enterobacteriaceae* in the tested maize silages (MS) were low, with only 3% of samples containing more than 10^2^ cfu/g. Even lower contamination levels have been reported in Slovakia (less than 1 log cfu/g) and Sweden (ranging from below 1.0 to 2.0 log cfu/g) [114,115]. In contrast, the *Enterobacteriaceae* count in Lithuanian MS varied from 3.0 to 4.8, with a mean value of 3.93 log_10_ cfu/g [73]. The observed *Enterobacteriaceae* burden suggests a low risk of foodborne pathogens in these forages, particularly since most members of this family are saprophytes. The genus *Salmonella* does not survive at a pH below 3.8 and is rarely found in silage. However, in Israel, both fresh plants and one out of five maize silages from sewage-irrigated fields tested positive for *Salmonella* [116]. Our survey did not detect *Salmonella* spp., despite the widespread use of organic fertilizers by farmers and the excessively high pH values found in one-third of the forages.

Since ruminants are recognized as a major reservoir of Shiga toxin-producing *E. coli* (STEC), and feeding on MS significantly increases the risk of enterohemorrhagic *E. coli* (EHEC) shedding among cattle, silages have been identified as a potential vehicle for the spread of STEC responsible for human illness [116,117]. A key feature of pathogenic *E. coli* strains is their acid resistance, which enables them to survive in fermented environments [118,119]. Of particular importance to the food production chain, including primary production, is the finding that once induced, these acid-resistance systems remain active during prolonged cold storage at 4 °C. In the present study, the total *E. coli* count in Polish MSs was generally below 10 cfu/g, except for approximately 2% of silages where contamination levels ranged from 1.3 to 4.7 log_10_ cfu/g. *E. coli* was detected more frequently in MSs with a correct pH than in those with an incorrect pH. However, it cannot be ruled out that this observation results from the limited number of *E. coli*-positive samples analyzed. In other studies on corn silage, *E. coli* populations were either eliminated during ensiling due to a rapid drop in pH or were found in decaying sections where the pH had increased [116,120]. A low total *E. coli* count implies an even lower likelihood of STEC or EHEC presence, as the vast majority of *E. coli* strains are commensal.

Aerobic spore-forming *Bacilli* are undesirable in MS and are typically associated with areas where the pH has not been sufficiently reduced or where aerobic deterioration has begun. *Bacillus* spores survive the ensiling process and passage through the intestinal tract of ruminants, subsequently contaminating milk via feces and causing significant issues for the dairy industry [121]. *Bacilli* are identified in silages more frequently than other groups of microorganisms, as they are common soil residents and part of the natural plant microbiome [7]. A particularly challenging species for the dairy industry is *B. cereus*, due to the ability of certain strains to produce enterotoxins. It grows optimally within a temperature range of 4 to 48 °C and a pH range of 4.5 to 9.5 under aerobic conditions in the presence of carbohydrates—conditions that may occur when silos are opened [122]. In our survey, approximately one quarter of MSs were *B. cereus*-positive. In other studies, the prevalence of *B. cereus* in MS ranged from occasional detection to 45% of samples [123,124]. The concentration of *B. cereus* spores in silage averaged 2.4 log_10_ spores/g, increasing by 0.4–0.5 log_10_ during the summer. In the Netherlands, concentrations ranging from 10^2^ to 10^6^ cfu/g were recorded, with high spore levels in the surface layers [124,125]. Similarly, a British study reported up to 10^5^ cfu/g, while a maximum of 200 spores/g was noted in Sweden [123,126]. Our results showed comparable levels, with a maximum of 3.4 log_10_ spores/g. According to the literature, *Bacillus* species isolated from silage include *B. cereus*, *B. licheniformis*, *B. pumilus*, *B. coagulans*, *B. sphaericus*, *B. polymyxa*, *B. lentus*, *B. firmus*, *B. sporothermodurans*, and *B. siralis* [7,30,121,127,128,129]. Our study also identified *B. weihenstephanensis*, *B. mycoides*, and *B. thuringiensis*—species closely related to *B. cereus*. *B. weihenstephanensis* and *B. mycoides* are psychrophilic cytotoxic bacteria, and *B. weihenstephanensis* causes spoilage of pasteurized milk [130,131]. Meanwhile, *B. thuringiensis* can cause food poisoning, infections, and bacteremia in humans, particularly in immunocompromised individuals [132].

Both coagulase-positive and coagulase-negative *Staphylococcus* species cause mastitis, therefore, understanding their distribution in dairy farms is important [133,134,135,136,137]. Although the silage environment is generally not conducive to the growth of *Staphylococcus* species, some studies have confirmed their presence, likely as secondary contaminants. Staphylococci are widely distributed in nature, commonly inhabiting the skin and mucous membranes of warm-blooded organisms [138]. In our study, we detected coagulase-negative *S. lentus*, which is known to cause infections in both humans and animals, as well as food poisoning [139,140]. Another survey showed the presence of the *Staphylococcus* genus at a level of 3.85% and *S. gallinarum* species at 1.48% among the bacterial microbiota of fermented feeds [141].

The abundance and diversity of the microbiome in the forage before ensiling are significantly higher than afterwards due to anaerobic fermentation and low pH which inhibit many microorganisms. Nevertheless, the high moisture of MS makes it a suitable environment for microbial growth. Hygienic indicators like TPC or AMB are used to asses sanitary quality, sensory acceptability, and conformance with good manufacturing practice. They provide information on the quality or handling history of input plants, ensiling, and storage conditions. Additionally, these indicators can be used to determine the shelf-life or forthcoming sensory change in silage. They estimate the likelihood of pathogen occurrence, including their harmful toxins, although there is no direct and accurate correlation to the biohazard burden. In interpreting AMB and TPC results of fermented feed such as silage, a high microbial load is expected. The TPC in our survey ranged from 1.6 to 9.7 log_10_ cfu/g in all samples tested. Other researchers recorded levels of 3.2–3.9 log_10_ cfu/g in Slovakia and 5.7–6.6 log_10_ cfu/g in Nigeria [142,143]. The AMB count in all Polish MSs was enumerated from 1.7 to 9.6 log_10_ cfu/g. The German MSs were loaded at 9.1–9.3 log_10_ cfu/g, and Brazilian samples had 5.35 log_10_ cfu/g [144,145]. Another German study found that nearly 47% of the sampled MSs from the North region achieved quality level 1, while almost 30% were assigned to quality level 4 according to reference ranges determined by the Association of German Agricultural Inspection and Research Institutes [69,146]. In the South region, the results were only slightly better, with 52.2% being assigned the best quality level (QS = 1) and 24.7% being assigned the worst quality level (QS = 4). In the East region, the microbiological quality of MSs was the best compared to other survey regions, although more than 10% of the forages were more or less spoiled. In turn, testing of MSs from all over Germany showed that from 47% to 73.5% of silages were of good, fault-free quality and were classified at quality level 1.

Ensiling is known to reduce fungal counts due to oxygen limitation [147]. A count of 4 log_10_ cfu/g is considered the limit recommended by good manufacturing practices in animal feed [4,148]. A fungi count below 4 log_10_ cfu/g indicates silage of good quality. Exceeding the reference value can affect palatability and reduce nutrient absorption, determining low hygienic quality and improper storage. It may cause respiratory problems, abnormal ruminal fermentation, decreased reproductive function, kidney damage, and skin and eye irritation. An action limit of 6 log_10_ cfu/g for molds is a feasible limit agreed upon in consultation with the sector, supplier, or customer. If this value is exceeded, an investigation must be conducted into the source of contamination, and measures must be taken to remove or limit it. The fungi count of Polish MSs ranged from 1 to 8.9 log_10_ cfu/g. A count below 4 log_10_ cfu/g was found in 78.3% of samples, while counts over 6 log_10_ cfu/g were found in 11.1% of forages. Fungal loads in other countries showed 2.1–5.1 log_10_ cfu/g in Brazil, 3.8–5.2 in Argentina, 4.3–4.5 log_10_ cfu/g in Canada, 1.6–3.1 log_10_ cfu/g in Italy, 5.3–6.8 log_10_ cfu/g in Germany, 5.8–6.0 in the Netherlands, and 3.5–7.1 log_10_ cfu/g in Lithuania [6,67,68,71,84,120,145,149,150]. These reports contradict the conventional presumption that MS from hot or tropical regions is usually of poorer quality compared to silage produced in cool or temperate climates due to uncontrollable climate-related factors [151,152].

The proper count of LAB in silage can reduce pathogenic bacteria and improve animal performance, including milk production, possibly due to the inhibition of detrimental microbes and interactions with rumen microbes [153,154]. The total LAB population in silage typically oscillates around 7 log_10_ cfu/g. A higher inoculation rate resulted in a lower total count observed [7,71]. Our survey demonstrated the LAB count from 1 to 9.3 log_10_ cfu/g in MSs from across the country. Nearly 75% demonstrated less than 6 log_10_ cfu/g, while more than 8 log_10_ cfu/g was shown in only 3.4% of MSs. A LAB count below 2 log_10_ cfu/g was evidenced in 13.5% of silages with a 2.6–4.4 pH value. Detected low levels of LAB in some forages might be a response of these bacteria to adverse environmental conditions, causing them to turn dormant [155]. Other researchers recorded LAB counts at 6.5–7 log_10_ cfu/g in Argentina, 6.8–8.7 log_10_ cfu/g in Brazil, 5.6–6.1 log_10_ cfu/g in Nigeria, 5.7 log_10_ cfu/g in Japan, 5.8–8.6 log_10_ cfu/g in Canada, 5.3–7.3 log_10_ cfu/g in Lithuania, and 4.1–5.1 log_10_ cfu/g in Slovakia [62,67,71,84,90,142,143,149,156,157]. Microbial inoculation of MS reduced variation in the ensiling process, affecting many pathogenic and deteriorative microorganisms and improving the quality of silage [37,120]. This finding was also evidenced in our study by the decrease in fungi, total bacteria, LAB, and *Enterobacteriaceae* counts, as well as the prevalence of *Listeria* spp., *Clostridium* spp., *C. perfringens*, the *Enterobacteriaceae* family, and *E. coli*.

## 5. Conclusions

Silage safety is a precondition for the safety of food of ruminant origin and human health, as well as a necessity for good animal health and welfare. While the proper ensiling process has the potential to reduce contamination of input plants, it is often unpredictable due to variation in epiphytic microorganisms and forage composition. Our study showed that a large share of microbial spoilage was related to high pH, and contamination with *Clostridium*, *B. cereus*, and fungi was significant. These observations confirm metabolic mechanisms exhibited by these microorganisms when the MS pH exceeds 4.5. Excessively high pH level, resulting from insufficient lactic acid production by LAB, allow *Clostridia* to dominate and initiate butyric fermentation. ASFB degrade amino acids, thereby inhibiting the pH decline and facilitating further *Clostridia* proliferation. Acid-tolerant yeasts metabolize sugars and organic acids; thus, the subsequent increase in pH and temperature triggers fungal growth. *B. cereus* germinates at pH level above 4.5 and due to its highly proteolytic nature, it stabilizes the alkaline environment, promoting the growth of other spoilage microorganisms, including pathogens. Hence, there is a need to prioritize fermented feed quality, specifically by minimizing crop contamination from soil and manure during harvesting, ensiling and storage. It also provides an incentive to test the silage for microbial status for effective cattle feeding, as a deficient quality of forages affects the health of animals. Every practical effort should be taken to eliminate contamination of silage, viewed as a critical point for the entry of pathogens into the food production chain. The sample size and microbial status of the MSs found in our study expand knowledge on hygiene of these forages and provide data to scientific risk assessment and risk management recommendations on the prevalence and count of microorganisms. Strictly anaerobic conditions of MS inhibit the proliferation of facultatively anaerobic pathogens, such as *E. coli*, *L. monocytogenes*, and *B. cereus*, within a pH range of 3.4 to 4.3. Simultaneously, anaerobic environment with as low as pH 4.1 allow for the growth of *C. butyricum*—a primary cause of butyric fermentation, and pH level above 4.5 facilitate the growth of botulinum toxin-producing *Clostridia*. The recommended limit for microbial indicators in MS is up to 2 cfu/g for both *Clostridia* and *Bacillus*, below 50 cfu/g for *C. perfringens*, and below 4 cfu/g for the total fungal count. Furthermore, the hygienic quality and health safety of silage are enhanced by inoculants, especially when there is a high risk of aerobic spoilage, a low epiphytic bacteria on maize at harvest, high moisture of the crop, or a high risk of fungal spoilage.

The authors acknowledge the impact of freezing on microorganism viability, yet this remained the only feasible approach for a national-scale study. Given the significant distances between sampling sites and the laboratory, refrigeration alone was insufficient. To ensure consistency, all silage samples were subjected to the same freezing protocol, meaning any potential changes in microbial counts were uniform across the entire dataset. A limitation of this study is the lack of food chain tracing for hazards originating in maize silage and their transmission through dairy cows to raw milk. This applies to both biological and chemical hazards, including mycotoxins, despite the high fungal contamination observed in some samples. Furthermore, the assessment of microbiological quality and pH levels remains incomplete without data on moisture content at harvest and organic acid concentrations in silage. Consequently, our future research will incorporate these parameters to provide a more comprehensive analysis.

## Figures and Tables

**Table 1:** Standard culture methods used in the study.

No and Title of Method		Step of Method				Detection/Quantification Limit
		Primary Enrichment	Secondary Enrichment	Isolation	Confirmation	
		liquid Non-Selective Medium/Incubation Conditions	Selective Liquid Medium/Incubation Conditions	Selective Agar Medium/Incubation Conditions	Test	
ISO 6579:2002 Horizontal method for the detection of *Salmonella* spp. [38]		Buffered Peptone Water ^1^/37 °C/18 h	Rappaport–Vassiliadis soya peptone broth (RVS) ^1^/41.5 °C/24 h	Xylose lysine deoxycholate agar (XLD) ^2^/37 °C/24 h	biochemical identification, serological identification	LOD ^5^ ≥ 3 cfu/g
			Muller–Kauffmann tetrathionate-novobiocin broth (MKTTn) ^2^/37 °C/24 h	Brilliant green agar (BGA) ^2^/37 °C/24 h		
ISO 4833-1:2013 Horizontal method for the enumeration of microorganisms. Part 1. Colony count at 30 degrees C by the pour plate technique [39]		NA	NA	Plate count agar (PCA) ^2^/30 °C/72 h	NA	LOQ ^6^ ≥ 10 cfu/g
PN-R-64791:1994 Animal feedingstuffs. Requirements and microbiological examination [40]	presence of anaerobic spore-forming bacteria (*Clostridium*)	NA	Wrzosek broth ^3^/37 °C/48 h/anaerobic conditions	Willis-Hobbs agar ^3^/37 °C/48 h/anaerobic conditions	biochemical identification, microscopic identification (Gram staining)	LOD < 10 cfu/g
				Wilson-Blair agar ^2^/37 °C/48 h/anaerobic conditions		
	enumeration of fungi	NA	NA	Dichloran-rose Bengal chloramphenicol agar (DRBC) ^2/^25 °C/7 d	NA	LOQ ≥ 10 cfu/g
	enumeration of aerobic mesophilic bacteria	NA	NA	Nutrient agar ^2/^37 °C/48 h	NA	LOQ ≥ 10 cfu/g
ISO 11290-1:1999/A1:2004 Horizontal method for the detection and enumeration of *Listeria monocytogenes*. Part 1. Detection method [41]		half-Fraser broth ^2^/37 °C/24 h	Fraser broth ^2^/37 °C/24 h	ALOA agar ^4/^37 °C/24 h	biochemical identification/	LOD ≥ 2 cfu/g
				Oxford agar ^2/^37 °C/24 h		
ISO 7937:2004 Horizontal method for the enumeration of *Clostridium perfringens*. Colony-count technique [42]		NA	NA	Tryptose sulfite cycloserine agar (SC) ^2/^37 °C/20 h/a anaerobic conditions	biochemical identification	LOQ ≥ 10 cfu/g
ISO 21528-2:2004 Horizontal method for the detection and enumeration of *Enterobacteriaceae*. Part 2. Colony-count technique [43]		NA	NA	Violet red bile glucose agar (VRBG) ^1/^37 °C/24 h	biochemical identification	LOQ ≥ 10 cfu/g
ISO 16649-2:2001 Horizontal method for the enumeration of beta-glucuronidase-positive *Escherichia coli*. Part 2. Colony-count technique at 44 degrees C using 5-bromo-4-chloro-3-indolyl beta-D-glucuronide [44]		NA	NA	Tryptone bile x-glucuronide agar (TBX) ^2/^44 °C/24 h	NA	LOQ ≥ 10 cfu/g
ISO 6888-2:1999 Horizontal method for the enumeration of coagulase-positive staphylococci (*Staphylococcus aureus* and other species). Part 2: Technique using rabbit plasma fibrinogen agar medium [45]		NA	NA	Baird-Parker RPF agar ^2/^37 °C/24–48 h	NA	LOQ ≥ 10 cfu/g
PN-A-82055-12 Detection of anaerobic spore-forming bacteria and anaerobic spore-forming *sulfate-reducing* bacteria (IV) [46]		NA	Wrzosek broth ^3^/37 °C/48 h/anaerobic conditions	Willis-Hobbs agar ^3^/37 °C/48 h/anaerobic conditions	biochemical identification, microscopic identification (Gram staining)	LOD < 10 cfu/g
				Wilson-Blair agar ^2/^37 °C/48 h/anaerobic conditions		
ISO 10272-1:2006 Horizontal method for detection and enumeration of *Campylobacter* spp. Part 1: Detection method [47]		NA	*Bolton* selective enrichment broth ^2^/41.5 °C/48 h/microaerobic conditions	Modified charcoal-cefoperazone-deoxycholate agar (mCCDA) ^2^/41.5 °C/48 h/microaerobic conditions	biochemical identification, morphological identification	LOD ≥ 5 cfu/g
ISO 15214:1998 Horizontal method for the enumeration of mesophilic lactic acid bacteria. Colony-count technique at 30 degrees C [48]		NA	NA	De Man, Rogosa and Sharpe agar (MRS) ^2^/30 °C/72 h	NA	LOQ ≥ 10 cfu/g
ISO 7932:2004 Horizontal method for the enumeration of presumptive *Bacillus cereus*. Colony-count technique at 30 degrees C [49]		NA	NA	Mannitol yolk polymyxin agar (MYP) ^2/^30 °C/24 h	biochemical identification	LOQ ≥ 10 cfu/g

NA—not applied, d—days, h—hours, ^1^ Merck KGaA, Darmstadt, Germany; ^2^ Oxoid Ltd., Basingstoke, UK; ^3^ BTL Sp. z o.o., Lodz, Poland; ^4^ Bio-Rad Laboratories, Inc., Hercules, CA, USA; ^5^ LOD—limit of detection; ^6^ LOQ—limit of quantification.

**Table 2:** Distribution of microbial counts (log_10_ cfu/g) in MSs of correct pH and descriptive statistics.

Microorganism	1 log_10_	2 log_10_	3 log_10_	4 log_10_	5 log_10_	6 log_10_	7 log_10_	8 log_10_	9 log_10_	Range (log_10_ cfu/g)	Mean Value (log_10_ cfu/g)	Median Value (log_10_ cfu/g)
	% of Samples											
TPC	0.4	1.4	9.1	35.1	30.1	14.9	6.5	1.8	0.4	1.6–9.4	5.3	5.0
AMB	0.7	2.9	12.3	46.4	23.6	6.9	4.3	1.4	1.1	1.8–9.5	4.9	4.7
Fungi	60.5	13.8	6.5	6.9	3.6	4.0	2.5	1.8	0	1–8.9	2.3	1.3
LAB	14.5	13.8	15.9	20.3	13.8	11.6	7.2	2.5	0	1–8.9	4.2	4.1
*Enterobacteriaceae*	97.5	0.7	0	1.1	0	0.4	0	0	0	1–6.2	1.1	1.0
*E. coli*	98.2	0.4	0.4	0.7	0	0	0	0	0	1–4.7	1.0	1.0
*Clostridium*	21.4	28.6	28.3	15.6	5.8	0	0	0	0	1–5	2.5	2.0
*C. perfringens*	97.1	2.5	0	0	0	0	0	0	0	1–2.6	1.0	1.0
*B. cereus*	87.3	11.6	0.7	0	0	0	0	0	0	1–3.4	1.2	1.0

**Table 3:** Distribution of microbial counts (log_10_ cfu/g) in MSs of incorrect pH and descriptive statistics.

Microorganism	1 log_10_	2 log_10_	3 log_10_	4 log_10_	5 log_10_	6 log_10_	7 log_10_	8 log_10_	9 log_10_	Range (log_10_ cfu/g)	Mean Value (log_10_ cfu/g)	Median Value (log_10_ cfu/g)
	% of Samples											
TPC	0	0.8	3.8	23.8	32.3	18.5	13.1	3.8	3.8	2.4–9.7	5.8	5.6
AMB	0	3.8	10.0	36.2	28.5	8.5	7.7	2.3	3.1	2.4–9.7	5.3	4.9
Fungi	47.7	16.9	7.7	4.6	5.4	7.7	6.9	3.1	0	1–8.6	3.0	2.0
LAB	11.5	10.0	11.5	13.1	20.8	17.7	8.5	6.2	0.8	1–9.3	4.8	5.0
*Enterobacteriaceae*	95.4	0.8	0.8	3.1	0	0	0	0	0	1–4.9	1.1	1.0
*E. coli*	99.2	0	0.8	0	0	0	0	0	0	1–3	1.0	1.0
*Clostridium*	13.8	35.4	30.8	12.3	7.7	0	0	0	0	1–5	2.6	3.0
*C. perfringens*	93.8	5.4	0	0.8	0	0	0	0	0	1–4.2	1.1	1.0
*B. cereus*	80.0	15.4	4.6	0	0	0	0	0	0	1–3.2	1.3	1.0

**Table 4:** Distribution of microbial counts (log_10_ cfu/g) in total MSs and descriptive statistics.

Microorganism	1 log_10_	2 log_10_	3 log_10_	4 log_10_	5 log_10_	6 log_10_	7 log_10_	8 log_10_	9 log_10_	Range (log_10_ cfu/g)	Mean Value (log_10_ cfu/g)	Median Value (log_10_ cfu/g)
	% of Samples											
TPC	0.2	1.2	7.4	31.5	30.8	16.0	8.9	2.5	1.5	1.6–9.7	5.5	5.2
AMB	0.5	3.2	11.6	43.3	25.1	7.4	5.4	1.7	1.7	1.8–9.7	5	4.8
Fungi	56.7	14.8	6.9	6.2	4.2	5.2	3.9	2.2	0	1–8.9	2.5	1.6
LAB	13.5	12.6	14.8	18.0	16.0	13.5	7.6	3.7	0.2	1–9.3	4.4	4.5
*Enterobacteriaceae*	97.0	0.7	0.2	1.7	0	0.2	0	0	0	1–6.2	1.1	1.0
*E. coli*	98.8	0.2	0.5	0.5	0	0	0	0	0	1–4.7	1.0	1.0
*Clostridium*	19.0	31.0	29.1	14.5	6.4	0	0	0	0	1–5	2.6	2.5
*C. perfringens*	96.3	3.4	0	0.2	0	0	0	0	0	1–4.2	1.1	1.0
*B. cereus*	85.0	13.1	2	0	0	0	0	0	0	1–3.4	1.2	1.0

**Table 5:** The differences in microbial load of inoculated and non-inoculated and organically fertilized and non-fertilized MSs.

Microorganism	Maize Silages											
	Inoculated			Non-Inoculated			Fertilized			Non-Fertilized		
	Range (log_10_ cfu/g)	Mean Value (log_10_ cfu/g)	Median Value (log_10_ cfu/g)	Range (log_10_ cfu/g)	Mean Value (log_10_ cfu/g)	Median Value (log_10_ cfu/g)	Range (log_10_ cfu/g)	Mean Value (log_10_ cfu/g)	Median Value (log_10_ cfu/g)	Range (log_10_ cfu/g)	Mean Value (log_10_ cfu/g)	Median Value (log_10_ cfu/g)
TPC	1.60–9.14	5.38	5.07	2.59–9.71	5.52	5.25	1.60–9.54	5.43	5.19	2.43–9.71	5.57	5.17
AMB	1.77–9.20	4.94	4.79	2.41–9.66	5.10	4.85	1.84–9.51	5.08	4.86	1.77–9.66	4.90	4.72
Fungi	1–8.47	2.53	1.47	1–8.89	2.57	1.69	1–8.89	2.43	1.47	1–8.60	2.91	1.77
LAB	1–9.27	4.30	4.07	1–8.90	4.50	4.63	1–8.90	4.33	4.43	1–9.27	4.69	4.63
*Enterobacteriaceae*	1–6.23	1.07	1.0	1–4.86	1.09	1.0	1–6.23	1.08	1.0	1–4.07	1.08	1.0
*E. coli*	1–4.71	1.03	1.0	1–4.14	1.03	1.0	1–4.71	1.03	1.0	1–4.14	1.04	1.0
*Clostridium*	1.0–5.0	2.53	2.0	1.0–5.0	2.61	3.0	1.0–5.0	2.60	3.0	1.0–5.0	2.51	2.0
*C. perfringens*	1–2.43	1.05	1.0	1–4.23	1.08	1.0	1–2.69	1.06	1.0	1–4.23	1.10	1.0
*B. cereus*	1–3.39	1.26	1.0	1–3.17	1.18	1.0	1–3.39	1.24	1.0	1–3.17	1.14	1.0

## Data Availability

The original contributions presented in this study are included in the article. Further inquiries can be directed to the corresponding author.
